# Recombinant interferon in advanced breast cancer.

**DOI:** 10.1038/bjc.1984.96

**Published:** 1984-05

**Authors:** A. Nethersell, H. Smedley, M. Katrak, T. Wheeler, K. Sikora

## Abstract

Fifteen patients with locally advanced refractory breast cancer have been treated with recombinant leucocyte interferon ( rIFN -alpha A) for up to 12 weeks. Toxicity was considerable with the initial dosage schedule employed but became acceptable after reducing the starting dose by 50%. Minor side effects occurred in all patients and major CNS toxicity in six. Nine patients showed some evidence of tumour regression at 4 weeks. Only two of these were still responding at 12 weeks. Response was unrelated to the length of previous history, oestrogen receptor status or previous responsiveness to cytotoxic or hormone therapy.


Br. J. Cancer (1984), 49, 615–620

### Recombinant interferon in advanced breast cancer

A. Nethersell, H. Smedley, M. Katrak, T. Wheeler & K. Sikora

*Ludwig Institute for Cancer Research, Department of Radiotherapy, Addenbrooke's Hospital, Cambridge; and Hinchingbrooke Hospital, Huntingdon, UK.*

**Summary** Fifteen patients with locally advanced refractory breast cancer have been treated with recombinant leucocyte interferon (rIFN-αA) for up to 12 weeks. Toxicity was considerable with the initial dosage schedule employed but became acceptable after reducing the starting dose by 50%. Minor side effects occurred in all patients and major CNS toxicity in six. Nine patients showed some evidence of tumour regression at 4 weeks. Only two of these were still responding at 12 weeks. Response was unrelated to the length of previous history, oestrogen receptor status or previous responsiveness to cytotoxic or hormone therapy.

Interferon has been the subject of scientific interest for over 20 years, first as an antiviral agent and later as an anticancer drug. Early preparations were produced by challenging cells in tissue culture with inducing agents such as viruses or synthetic RNA (Gresser, 1961). Three main cell types were used. Leucocytes derived from buffy coats, or from leukopheresis preparations, produced crude α-interferon of 0.1% purity which could be partially refined by precipitation methods (Cantell *et al.*, 1981; Horowitz, 1981). Human lymphoblastoid cell lines derived from patients with Burkitt's lymphoma resulted in a mixture of α and γ-interferon (Strander *et al.*, 1975). Normal fibroblasts grown in tissue culture resulted in β-interferon of higher specific activity (Giard *et al.*, 1979). It has gradually become clear that there are at least 3 gene families of inferferons: α, mainly derived from leucocytes, β from fibroblasts, and γ-interferon produced by mitogen or antigen stimulated lymphocytes.

With the advent of the recombinant DNA technology it is possible to produce vast quantities of interferon of high specific activity by genetic engineering. Human interferon genes have been isolated and cloned in bacterial plasmids (Goeddel *et al.*, 1980). The recombinant plasmids are inserted into *E. coli*, where they can replicate and produce mRNA, which in turn causes the bacterial protein synthesis machinery to make human interferon. Clones of *E. coli* have been isolated which produce large amounts of interferon. Using industrial fermentation techniques, unlimited quantities of interferon are now available for clinical use. There may be biochemical differences, such as the absence of glycosylation, between recombinant and natural interferons but the significance of these differences is not known.

Correspondence: K. Sikora, Ludwig Institute for Cancer Research, Cambridge.

Received 8 September 1983; accepted 18 January 1984.

Until recently clinical studies have used crude interferons of low purity. Profound side effects were observed which were initially attributed to impurities (Armin *et al.*, 1983). Furthermore, the inadequate supplies and difficulties in purification allowed only limited amounts of interferon to be used. Nevertheless there was a wealth of data showing regression of animal tumours (Gresser, 1977) and cytotoxic activity against tumour cell lines (Gresser, in press). In addition there were encouraging reports of tumour regression in patients with myeloma, non-Hodgkin's lymphoma, breast cancer, melanoma, renal carcinoma and Kaposi's sarcoma (Sikora, 1983). In patients with advanced breast cancer, 30% partial remission rates were reported by two groups using crude buffy coat interferon (Gutterman *et al.*, 1980; Borden *et al.*, 1980).

These preliminary results, together with the availability of large amounts of highly purified recombinant interferon, led us to set up a phase II study to look at the use of one preparation, recombinant leucocyte A interferon (rIFN-αA, Hoffman-La Roche) in patients with advanced breast cancer.

### Patients and methods

Fifteen patients with readily assessable disease were studied. Those with CNS metastases, malignant pleural effusions, ascites, blastic or mixed lytic/blastic osseous metastases as the only evidence of disease were excluded. Also excluded were patients with hypercalcaemia or impaired haematological, renal or hepatic function. Treatment with non-steroidal anti-inflammatory agents was not permitted during the study, Corticosteroid doses were no greater than physiological replacement. No anti cancer medication (hormones or chemotherapy) was given for the 4 weeks preceding the study. The dose of rIFN-αA was randomised to $20 \times 10^6$ units $m^{-2}$ daily or $50 \times 10^6 m^{-2}$ 3 times weekly by deep i.m. injection for 12 weeks. It was initially planned to study 60 patients but the study was stopped at 15 in view of the poor response rate. This dose was based on previously reported phase I studies (Gutterman *et al.*, 1982). Dose reductions to 50% and then 10% of the above doses were made in patients with toxic side effects. All patients were aware of their diagnosis and gave written informed consent to the study.

Patients were assessed on entry by full clinical examination, chest X-ray, bone scan, liver function tests, and photography of skin lesions. These investigations were repeated periodically during IFN therapy. Oestrogen receptor status was also determined on a repeat biopsy, performed under local anaesthetic in all patients. Patients were admitted to hospital for initiation of IFN therapy but in most cases were discharged after several days. A community research nurse continued outpatient treatment and provided a crucial link between the patient's home and the clinic. Patients were formally assessed by two of us at regular intervals for signs of tumour regression, toxicity and performance status. A disease marker, such as a lymph node or patch of skin infiltration which could be measured, was chosen as an indicator for response. The following criteria were used: (CR) for disappearance of all tumour; partial response (PR) for 50% reduction in the product of the longest perpendicular diameters; stable disease where no change occurred; or progressive disease when tumour growth continued unabated. We also used the term less than partial response (LPR) for a response not satisfying the criterion for PR. Clinical photographs were taken frequently since the measurement of extensive infiltrative or ulcerative chest wall disease by the above criteria was often difficult.

Toxicity was graded as in Table II and dose reductions made to 50% of the planned dose for two or more minor toxicities; to 10% if moderate toxicity was observed. The appearance of a major toxic effect resulted in the discontinuation of IFN until the effect disappeared.

### Results

The ages of the 15 patients ranged between 41 and 69 (mean 60). All were post menopausal and all had received previous treatment with one or more of the following: hormones, radiation, cytotoxic therapy (combination therapy or oral cyclophosphamide). All had progressive disease at the time of entry. Four had lymph node recurrences and the remaining eleven more widespread chest wall disease which was fungating or ulcerated in 4 patients. Three patients had pleural effusions and one pulmonary secondaries. Ten patients had positive bone scans.

In the first 11 patients treated, drastic dose reductions to 10% of the initial dose were necessary in all but one on account of lethargy and CNS toxicity. In the last 4 patients whose starting dose was reduced by 50% ($10 \times 10^6$ units $m^{-2}$ daily or $25 \times 10^6$ units $m^{-2}$ 3 times weekly) only one further small dose reduction was necessary in one patient. There were no obvious differences in toxicity between daily or 3 times weekly administration.

Four patients died during IFN therapy. In 2 of these death was possibly related to the treatment, either resulting from direct CNS toxicity or secondary to dehydration and renal failure which followed as a result of impaired cerebration. Two deaths occurred in patients with very advanced carcinomatosis and were considered disease-related.

### *Side effects*

*CNS* All patients experienced varying degrees of lethargy, anorexia, nausea, vomiting, headache and myalgia. Five developed transient paraesthesia. Severe lethargy preceded a dose related state of confusion, disorientation, and dysphasia, if regular IFN administration continued. Subsequent coma developed in the two patients whose deaths were possibly related to IFN therapy. No predisposing metabolic cause could be found for these deaths and there was no evidence of cerebral metastases. Severe CNS side effects were seen in 6 patients (Smedley *et al.*, 1983). Serum electrolytes, glucose and liver function tests remained unchanged during this syndrome. EEGs showed excess slow wave activity and in subsequent patients serial EEGs showed increasing slow wave activity as interferon therapy progressed despite the absence clinically of major CNS toxicity. The EEG abnormalities improved slowly after stopping treatment in the patients who have been reassessed at this stage. All four patients treated on the reduced dosage developed EEG slow wave changes although none developed major CNS toxicity. CNS toxicity appeared, therefore, to be dose related. There was no major toxicity for doses $< 10 \times 10^6$ units $m^{-2}$ daily.

*Weight loss* Nearly all patients lost weight and in some this was 10% or more of body wt. Weight loss resulted from anorexia, nausea, vomiting as well as the total loss of interest in sustenance and self preservation during periods of extreme lethargy and fatigue.

*Haematological* Nine patients showed a small fall in haemoglobin levels at 4 weeks. All but 2 patients showed a fall in the white cell count which occurred within the first 3 weeks and thereafter returned to near normal levels. The mean count fell from $6.6 \times 10^9 l^{-1}$ initially to $2.9 \times 10^9 l^{-1}$. There were no infections resulting from leukopenia and a neutrophilia occurred in two patients in response to infection (one chest infection and one case of cystitis). The platelet count fell in parallel with the white cell count, mean values falling from $315 \pm 101 \times 10^9 l^{-1}$ to $178 \pm 100 \times 10^9 l^{-1}$. Two patients who had received extensive chemotherapy developed counts below $100 \times 10^9 l^{-1}$ but there were no purpuric or haemorrhagic complications.

*Hepatic* The bilirubin and alkaline phosphatase levels did not appreciably alter during IFN therapy except in patients with progressive disease. The SGPT (L-alanine, 2-oxogluterate aminotransferase) rose in the second to third week in all patients and then returned slowly to normal. It appeared unrelated to major CNS toxicity and its significance is unknown (Table I).

**Table I** Mean SGPT levels (iu.$l^{-1}$)

| | |
|---|---|
| Initial | $24 \pm 8$[a] |
| Week 1–2 | $62 \pm 23$ |
| Week 3–4 | $35 \pm 14$ |
| Week 6–12 | $28 \pm 7$ |

[a]s.d.

*Fever* All patients developed pyrexia shortly after commencing IFN. This was easily controlled by paracetamol and settled within one week.

In order to assess the severity of the side effects we have classified them as major, moderate and mild (Table II) and recorded the proportion of time during IFN therapy that each patient suffered these side effects. Finally we have averaged the percentages over all patients (Table III). It is clear that by reducing the starting dose to $10 \times 10^6$ units $m^{-2}$ daily major toxicity was avoided, but that one-third of patients still had moderate, and two-thirds mild toxicity. The absence of a "no toxicity" category in this group is due to the fact that at this dose IFN therapy could continue whereas for the first 11 patients the treatment was frequently reduced to $3 \times 10^6$ units daily (or equivalent) after about 4 weeks, or stopped temporarily, and not restarted untill all toxic side effects had completely subsided.

We also looked at the time spent in hospital during IFN therapy. Ten patients were able to go home 2 days after starting treatment and only 3 of these needed readmission. The average time spent in hospital was 13 days with a range from 2 to 45 days. The Karnofsky status was assessed weekly and the median value of 90 (range 70–100) prior to IFN fell to 60 (range 30–80) during therapy.

**Table II** Toxicity of interferon

(*One symptom alone is sufficient to qualify for appropriate category*)

| | |
|---|---|
| Major: | Confusion<br>Disorientation<br>Dysphasia<br>Coma |
| Moderate: | Severe lethargy: sleeping up to 20 h per day<br>Severe anorexia<br>Severe nausea<br>Intractable vomiting |
| Mild: | Lethargy<br>Anorexia<br>Nausea and vomiting<br>Pyrexia<br>Headaches<br>Myalgia<br>Parasthesia |

**Table III** Time spent with each of the above toxicities expressed as a percentage of the total time receiving IFN

| | *No toxicity* | *Mild toxicity* | *Moderate toxicity* | *Major toxicity* |
|---|---|---|---|---|
| Patients 1–11 | 7% | 59% | 29% | 5% |
| Patients 12–15 | 0 | 68% | 32% | 0 |

### *Tumour response*

In the assessment of any new form of cancer treatment the odds are loaded heavily against the new modality in that patients must, for ethical reasons, have very advanced disease. Despite the severe toxicities encountered, we found that many patients were reluctant to stop therapy. Tumour regression was seen in lymph nodes, skin infiltration and ulcerated and fungating lesions. Arm lymphoedema also lessened on objective measurement. Analgesic requirements decreased in 3 patients with painful bone metastases and in one of these the serum alkaline phosphatase level fell. We also noted a phenomenon observed in other studies – regression of disease at one site with progression elsewhere.

Of the 12 patients assessed at 4 weeks, 2 were considered to have had true partial responses, and 7 less than partial responses. One patient had stable disease and one had disease progression. The remaining patient had a partial response at the

**Table IV**

Patient characteristics and response data

| Patient | Age | ER status | Time to 1st relapse (months) | Time from relapse to IFN (months) | Previous response to: Chemo | Previous response to: Hormones | Dose of IFN per week: Weeks 1–4 | Dose of IFN per week: Subsequent | Weeks on IFN | Survival from start of IFN (weeks) | Alive or dead | Major CNS toxicity | Response at 2 (weeks) | Response at 4 (weeks) | Response at 12 (weeks) |
|---|---|---|---|---|---|---|---|---|---|---|---|---|---|---|---|
| 1 | 60 | POS | 48 | 72 | Yes | Yes | 239 | 116 | 5 | 5 | D | + | S | LPR | — |
| 2 | 58 | NEG | 4 | 9 | NT | No | 175 | 0 | 4 | 65 | A | – | S | Prog[c] | — |
| 3 | 60 | POS | 49 | 22 | NT | Yes | 252 | 65 | 12 | 40 | D | + | S | PR | Prog[c] |
| 4 | 69 | POS | [a] | 46[b] | NT | Yes | 217 | 258 | 12 | 67 | A | – | LPR | PR | LPR |
| 5 | 41 | NEG | 2 | 12 | Yes | No | 252 | 0 | 2 | 2 | D | – | S | — | — |
| 6 | 54 | NEG | 7 | 6 | No | LPR | 162 | 59 | 12 | 30 | D | – | S | LPR | Prog |
| 7 | 66 | NEG | 16 | 1 | NT | No | 71 | 21 | 12 | 60 | A | + | LPR | LPR | Prog |
| 8 | 61 | NEG | 9 | 11 | Yes | No | 111 | 60 | 12 | 16 | D | + | S | LPR | Prog |
| 9 | 62 | NEG | 53 | 13 | No | No | 258 | 0 | 2 | 2 | D | – | S | — | — |
| 10 | 59 | NEG | 60 | 68 | Yes | Yes | 252 | 0 | 2 | 2 | D | + | S | — | — |
| 11 | 62 | NEG | [a] | 10[b] | LPR | LPR | 150 | 21 | 11 | 40 | D | + | S | S | Prog |
| 12 | 64 | NEG | 10 | 8 | No | No | 126 | 126 | 12 | 20 | D | – | LPR | LPR | Prog |
| 13 | 47 | POS | 16 | 6 | NT | No | 150 | 150 | 10 | 26 | A | – | S | LPR | Prog |
| 14 | 67 | NEG | [a] | 5[b] | NT | No | 126 | 126 | 12 | 22 | A | – | S | LPR | LPR |
| 15 | 68 | POS | 51 | 51 | LPR | LPR | 92 | 0 | 4 | 12 | A | – | S | Prog | — |

[a]Patient presented with advanced local disease and never attained CR

[b]Measured from date of presentation

[c]Disease in opposite breast appeared although marker site responded

PR Partial response

LPR Less than partial response

Prog Disease progression

NT Not tried

NA Not assessable

S Stable disease indicator site but developed on going disease in the opposite breast. At 12 weeks, or cessation of therapy, 10 patients were alive. There were only 2 who showed any degree of response at this stage, the others having relapsed. It should be noted, however, that the dose of IFN administered for the last 8 weeks in most patients was much lower than for the first month.

We have explored whether there were any obvious predisposing factors governing response (Table IV). Here we tabulate age, oestrogen receptor (ER) status and the number of months from presentation to first relapse as well as the time between this relapse and starting IFN therapy. This indicates the natural history and aggressiveness of the disease process for each patient. We have also summarised response to previous cytotoxic or hormone therapy in an attempt to see whether any patterns of responsiveness emerge.

Other independent variables governing response are the dose of IFN used and the duration of therapy. Many patients had a dose reduction after 4 weeks. We have stated the *mean* dose of IFN given per week during the first 4 weeks and the subsequent weeks respectively for each patient. The responses at 4 and 12 weeks are presented as well as survival.

### Discussion

Response could not be related to the total duration of previous history, previous response to hormones or cytotoxic drugs, oestrogen receptor status or the dose of IFN administered. In the two patients who were still responding at 12 weeks, two factors emerge. Firstly, each patient had very advanced local disease without obvious metastases. The first patient had disease for 46 months from diagnosis and probably for 3 years prior to this. She had shown some improvement with tamoxifen. Clearly she had lived in symbiosis with her disease for a long time. The second patient had presented with a locally fungating carcinoma which she admitted having had for well over a year and we suspect much longer. There had been little response to 5 months tamoxifen therapy but equally little progression. In both cases the relative indolence of the disease could have been a function of the disease itself (i.e. mitotic rate) or of immunological factors in the host, or both. It is interesting, however, that both these patients showed the most consistent and sustained response to IFN. Little is known of the possible antitumour activity of IFN *in vivo*. Possible mechanisms include the inhibition of DNA-replication in the malignant cell following membrane binding or stimulation of the host's immune system.

**Table V** Average weekly dose rIFN-αA given during study

| | *First month* | *Subsequent* |
|---|---|---|
| Average for patients 1–11 | 194±62 | 86±76 |
| Average for patients 12–15 | 123±21 | 134±11 |
| Value for patient 4 | 217 | 258 |
| Value for patient 14 | 126 | 126 |

The second feature common to these two patients is that they completed 12 weeks of IFN treatment with relatively few toxic side effects and consequently with little dose reduction. To explore this further we have calculated the average weekly doses of IFN for the groups of patients 1–11 and 12–15 for the first month of IFN therapy, and subsequently, and compared these with the two responding patients (Table V). There was a marked dose reduction after one month owing to toxicity in the first 11 patients and this was overcome by reducing the starting dose. Patient 4 tolerated a dose which was far higher than the 12 week average for the other patients, and tumour regression may have been dose related. The dose of patient 14 was no different from the other 3 patients in her group who failed to respond, and not very different from the dose used in many of the first 11 patients. It seems unlikely, therefore, that her response was dose related. Patient 4 went on to maintenance IFN for one year, $86 \times 10^6$ units twice weekly. She has suffered no appreciable toxicity but has required additional treatment in the form of local irradiation and oral cytotoxic therapy. The response of her tumour to radiation has been excellent. Her EEG has showed persistent slow wave activity but has not significantly altered over the year.

The study would suggest that there is little place for rIFN-αA as a single agent in breast cancer although this study did not investigate its role in combination with other agents or as an adjuvant. There is no doubt, however, that many patients showed signs of tumour destruction at 4 weeks. The objective responses seen however encourage further study to investigate the potential value of IFN and its mechanism of action which are currently unknown. We also noted several responses to irradiation which were unexpectedly good in patients who were receiving, or had recently received, IFN. We are currently looking into this possible synergism, encouraged by the fact that others have made similar observations in relation to chemotherapy and interferon.

We thank Drs S. De Garis, Z. Dziewanowska, and I. Lenox-Smith of Hoffmann-La Roche for their helpful advice, Hoffman-La Roche for providing the interferon, and our colleagues for referring patients for this study.

### References

ARMIN, H.R., COURTNEY-McGREGOR, W. & DZIEWANOWSKA, Z. (1983). Methods of preparation. In: *Interferon and Cancer* (Ed. Sikora) New York, Plenum Press, 17.

BORDEN, E., DAO, T., HOLLAND, J., & 0 others. (1980). Interferon in recurrent breast carcinoma: preliminary report for the American Cancer Society Clinical Trials Program. *Proc. Am. Soc. Clin. Oncol.*, **21**, 187.

CANTELL, K., HIRVONEN, S. & KOISTINEN, V. (1981). Partial purification of human leukocyte interferon on a large scale. *Meth. Enzymol.*, **78**, 499.

GIARD, D.J., LOEB, D.H., THILLY, W.G., WANG, D.I.C. & LEVINE, D.W. (1979). Human interferon production with diploid fibroblast cells grown on microcarriers. *Biotechnol. Bioeng.*, **21**, 433.

GOEDDEL, D.V., YELVERTON, E., ULLRICH, A. & 15 others. (1980). Human leukocyte interferon produced by *E. coli* is biologically active. *Nature*, **287**, 411.

GRESSER, I. (1961). Production of interferon by suspension of human leukocytes. *Proc. Soc. Exp. Biol. Med.*, **108**, 799.

GRESSER, I. (1977). Cancer – a comprehensive treatise. *Chemotherapy*, **5**, 521.

GESSER, I. (in press). How does interferon inhibit tumour growth? *Phil. Trans. R. Soc.* (*Lond. B*).

GUTTERMAN, J., BLUMENSCHEIN, G., ALEXIAN, R. & 0 others. (1980). Leukocyte interferon – induced tumor regression in human metastatic breast cancer, multiple myeloma, and malignant lymphoma. *Ann. Intern. Med.*, **93**, 299.

GUTTERMAN, J. FEIN, S., QUESADA, J. & 00 others. (1982). Recombinant human leukocyte interferon (IFL-rA). A phase I clinical study of pharmacokinetics, single dose tolerance and biologic effects in cancer patients. *Ann. Intern. Med.*, **96**, 549.

HOROWITZ, B. (1981). Human interferon-properties, clinical application, and production. *J. Parenteral Sci. Technol.*, **35**, 223.

SIKORA, K. (Ed.) (1983). *Interferon and Cancer*, New York, Plenum Press.

SMEDLEY, H., KATRAK, M., SIKORA, K. & WHEELER, T. (1983). Neurological effects of recombinant human interferon. *Br. Med. J.*, **286**, 262.

STRANDER, H., MORGENSEN, K.E. & CANTELL, K. (1975). Production of human lymphoblastoid interferon. *J. Clin. Microbiol.*, **116**, 117.


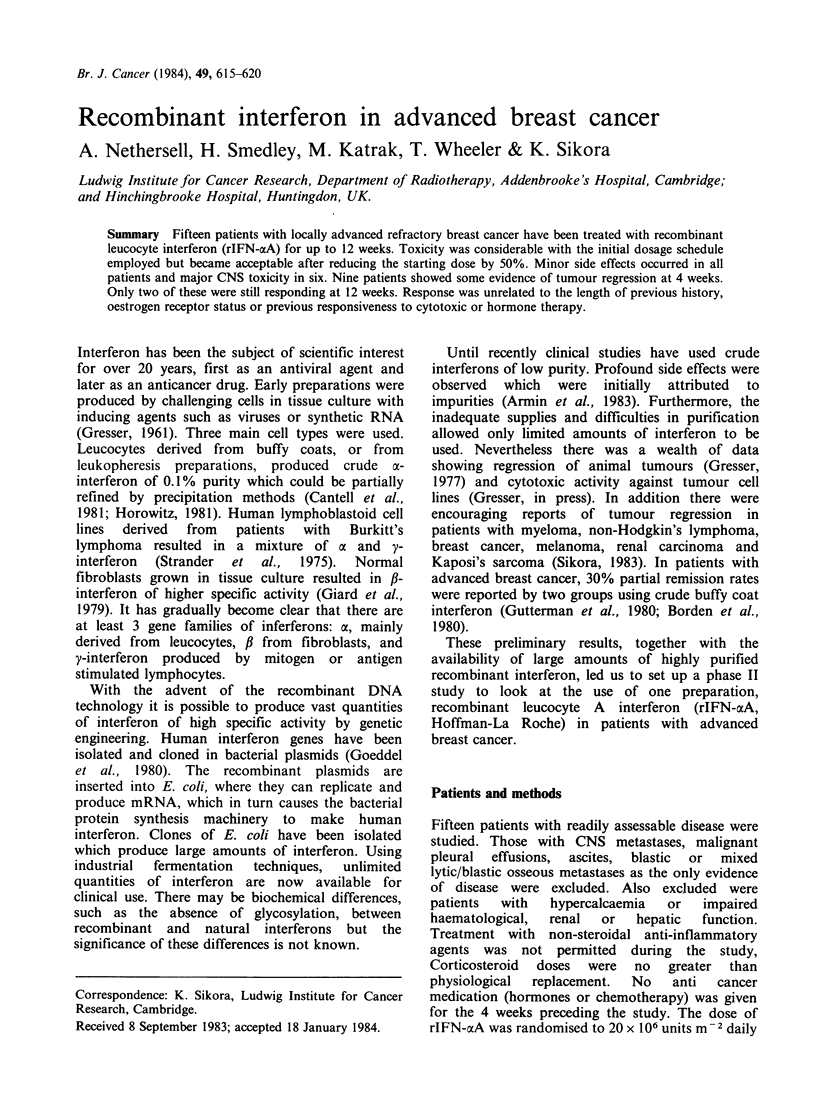

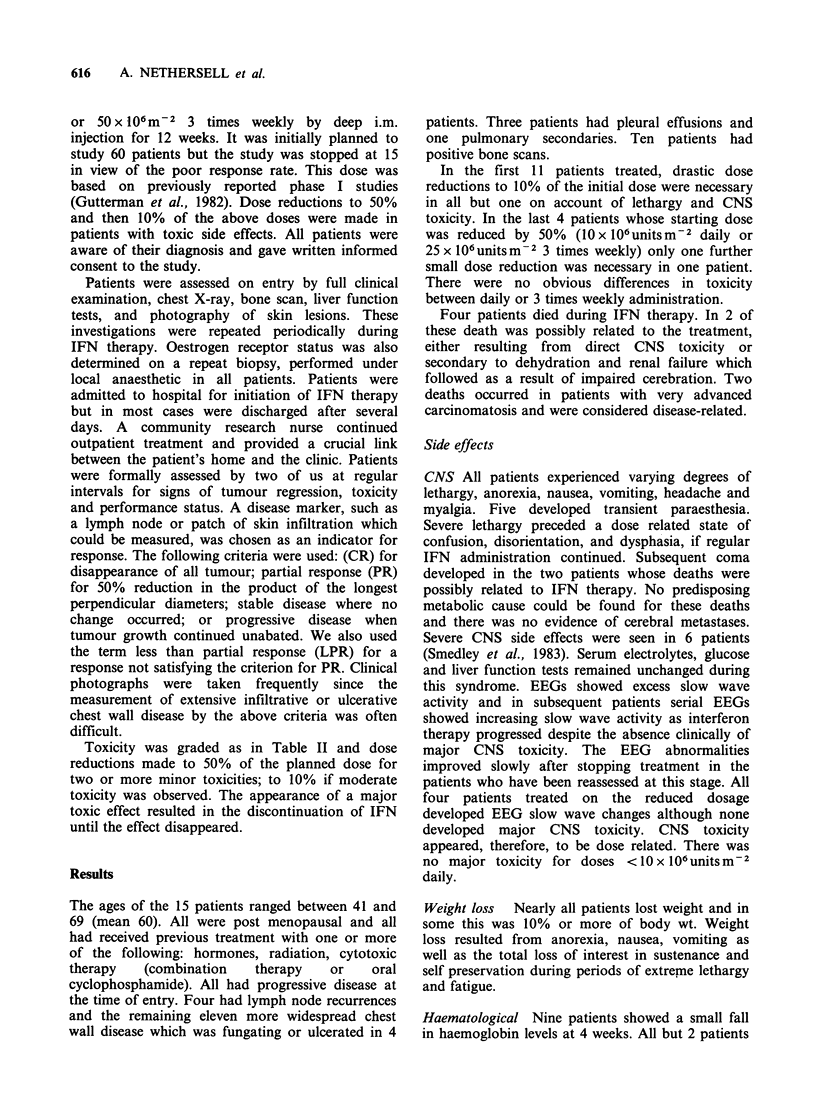

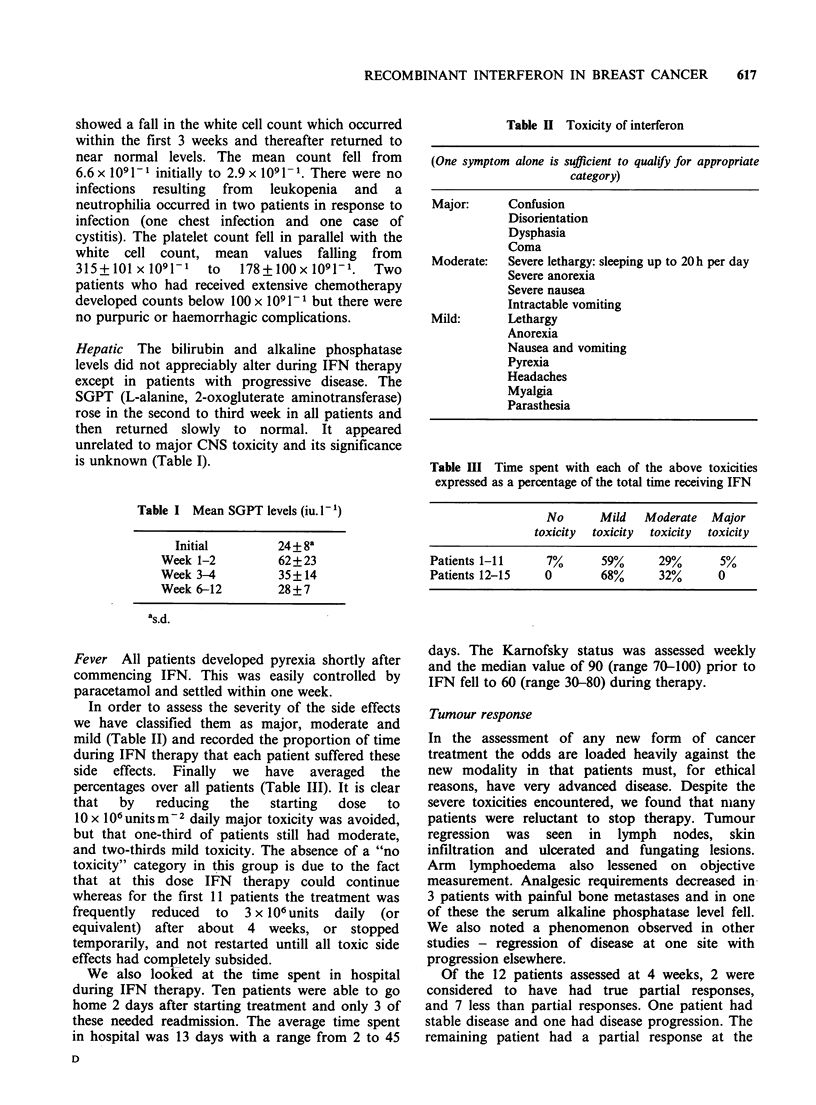

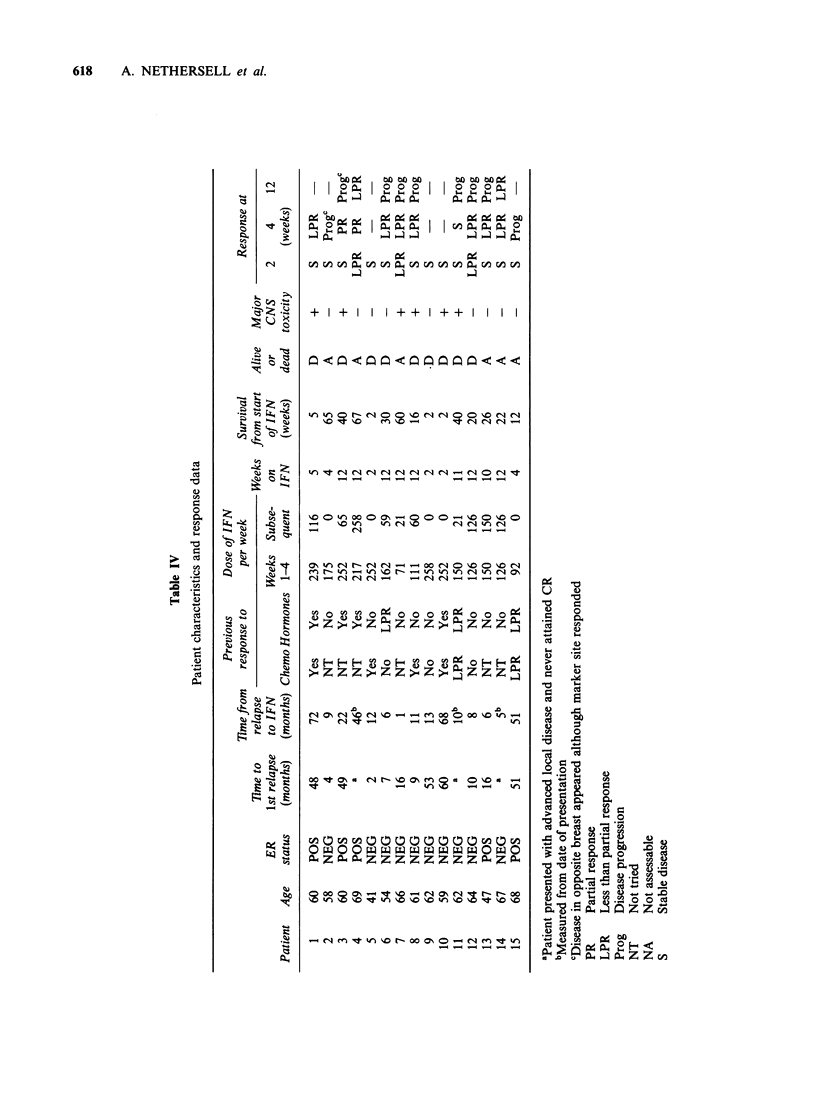

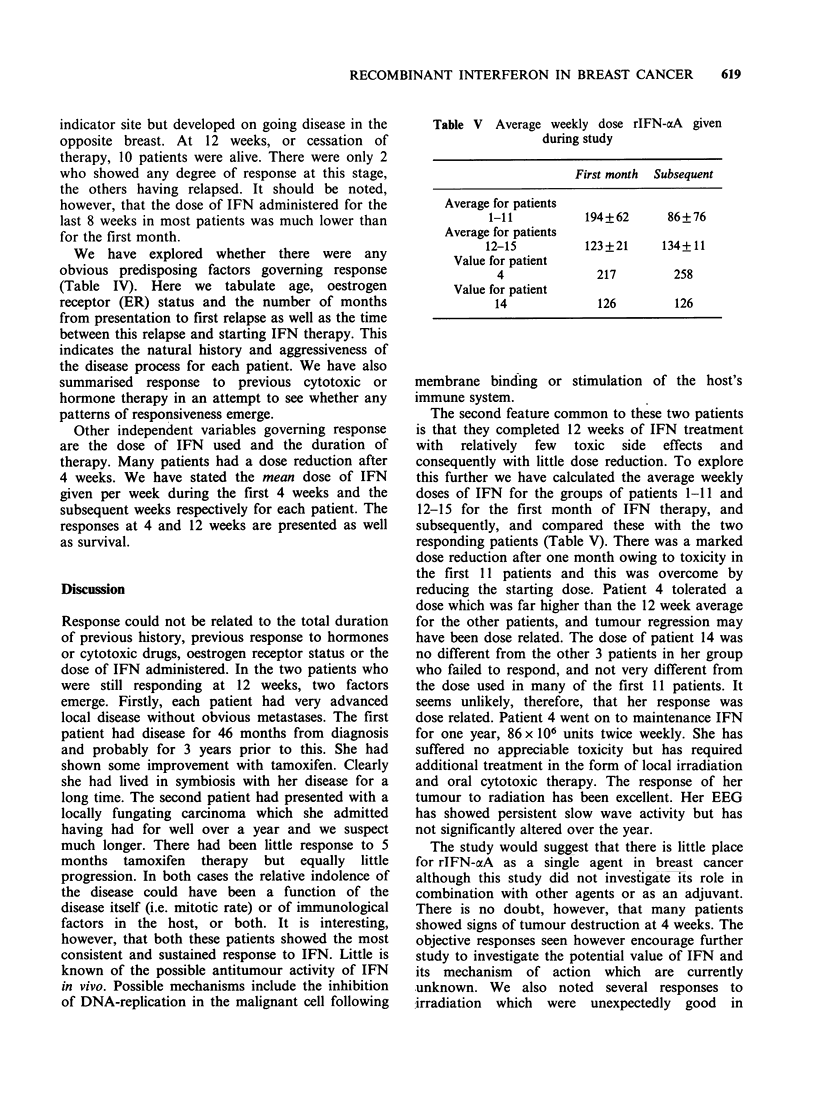

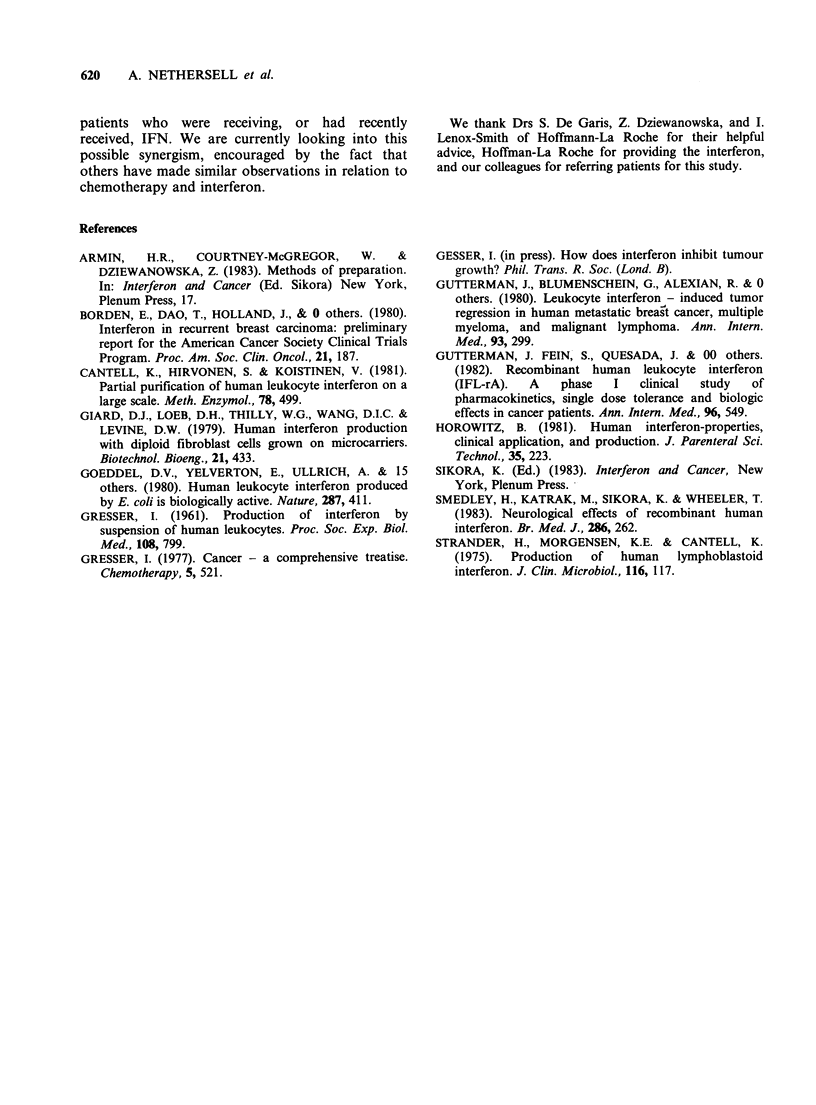


## References

[OCR_00616] Cantell K., Hirvonen S., Koistinen V. (1981). Partial purification of human leukocyte interferon on a large scale.. Methods Enzymol.

[OCR_00632] GRESSER I. (1961). Production of interferon by suspensions of human leucocytes.. Proc Soc Exp Biol Med.

[OCR_00621] Giard D. J., Loeb D. H., Thilly W. G., Wang D. I., Levine D. W. (1979). Human interferon production with diploid fibroblast cells grown on microcarriers.. Biotechnol Bioeng.

[OCR_00627] Goeddel D. V., Yelverton E., Ullrich A., Heyneker H. L., Miozzari G., Holmes W., Seeburg P. H., Dull T., May L., Stebbing N. (1980). Human leukocyte interferon produced by E. coli is biologically active.. Nature.

[OCR_00659] Horowitz B. (1981). Human interferon--properties, clinical application, and production.. J Parenter Sci Technol.

[OCR_00668] Smedley H., Katrak M., Sikora K., Wheeler T. (1983). Neurological effects of recombinant human interferon.. Br Med J (Clin Res Ed).

[OCR_00673] Strander H., Mogensen K. E., Cantell K. (1975). Production of human lymphoblastoid interferon.. J Clin Microbiol.

